# Effects of Fertilization and Clipping on Carbon, Nitrogen Storage, and Soil Microbial Activity in a Natural Grassland in Southern China

**DOI:** 10.1371/journal.pone.0099385

**Published:** 2014-06-10

**Authors:** Zhimin Du, Yan Xie, Liqun Hu, Longxing Hu, Shendong Xu, Daoxin Li, Gongfang Wang, Jinmin Fu

**Affiliations:** 1 Key Laboratory of Plant Germplasm Enhancement and Specialty Agriculture, Wuhan Botanical Garden, Chinese Academy of Sciences, Wuhan, Hubei, China; 2 Graduate University of Chinese Academy of Sciences, Beijing, Hebei, China; 3 National Dalaoling Forest Park, Yichang, Hubei, China; Wuhan Botanical Garden, Chinese Academy of Sciences, Wuhan, China, China

## Abstract

Grassland managements can affect carbon (C) and nitrogen (N) storage in grassland ecosystems with consequent feedbacks to climate change. We investigated the impacts of compound fertilization and clipping on grass biomass, plant and soil (0–20 cm depth) C, N storage, plant and soil C: N ratios, soil microbial activity and diversity, and C, N sequestration rates in grassland *in situ* in the National Dalaoling Forest Park of China beginning July, 2011. In July, 2012, the fertilization increased total biomass by 30.1%, plant C by 34.5%, plant N by 79.8%, soil C by 18.8% and soil N by 23.8% compared with the control, respectively. Whereas the clipping decreased total biomass, plant C and N, soil C and N by 24.9%, 30.3%, 39.3%, 18.5%, and 19.4%, respectively, when compared to the control. The plant C: N ratio was lower for the fertilization than for the control and the clipping treatments. The soil microbial activity and diversity indices were higher for the fertilization than for the control. The clipping generally exhibited a lower level of soil microbial activity and diversity compared to the control. The principal component analysis indicated that the soil microbial communities of the control, fertilization and clipping treatments formed three distinct groups. The plant C and N sequestration rates of the fertilization were significantly higher than the clipping treatment. Our results suggest that fertilization is an efficient management practice in improving the C and N storage of the grassland ecosystem via increasing the grass biomass and soil microbial activity and diversity.

## Introduction

The grasslands in China cover an area of 3.92 million km^2^ and provide 9% to 16% of the total C in the world grasslands [1], [2], [3]. Concerns about global warming has increased an attention to understand the role of potential C and nitrogen (N) sink in grasslands in mitigating the emission of greenhouse gases (i.e. CO_2_ and N_2_O) [4]–[6]. The C and N sequestration in terrestrial ecosystems constitutes a major mitigation strategy against the global warming [7]. China's grasslands make an important contribution to the world C and N storage and may have significant effects on C and N cycles worldwide [2]. Natural grasslands of southern China cover an area of 79.58 million km^2^, and probably have a high yield owning to good hydrothermal conditions [8], which can be an important C and N pool.

The processes of C and N sequestration can be greatly affected by grassland managements [9], and good managements are critical for grasslands to enhance C and N sequestration [10]–[12]. Compound fertilizers or organic amendments affected grasslands C and N storage via increasing plant biomass [10], [13], [14]. Dersch and Böhm [15] reported that N, phosphorus (P), and potassium (K) fertilizers combined with farmyard manure application enhanced C storage to about 5.6 Mg ha^−1^ after 21 years in Australia. The N fertilization and cover cropping can increase soil organic C and total N by increasing the amount of plant residues returned to the soil [11], [16]. Similarly, the application of manure can increase soil organic C and total N levels [17], [18]. Clipping was found to affect the grassland C and N storage via reducing plant biomass [9] and changing grass species [19]. Particularly, the potentially dominant plants (i.e. usually larger than their neighbors) often lose a higher proportion of their biomass than their neighbors after clipping [9].

Soil microorganisms exert a dominant influence on the net C and N balance of terrestrial ecosystems by controlling soil organic matter (SOM) decomposition and plant nutrient availability [20], [21]. The grassland SOM mainly derived from roots, senescent leaves and stems of the vegetations [22]. The processes and functions of breakdown of the plants residues in soil are greatly impacted by soil microorganisms [23]. Agricultural managements can affect soil microorganisms' condition and ultimately affect the C and N cycling in ecosystems [24], [25]. Microbial populations were significantly increased in the soils amended with green manure throughout two-year experiment [26]. Soil microbial diversity and/or activity may be a sensitive indicator of ecosystem change, as it can be quickly affected by disturbances [27], [28]. Zhong and Cai [29] demonstrated that soil microbial diversity and average well color development (AWCD) which reflects total microbial activity [30] in the NPK treatment were increased in response to fertilization. Soil microbial biomass, populations and diversity were increased by optimum and balanced fertilization [31], [32]. On the other hand, the clipping significantly reduced soil microbial and respiration rate in both warmed and un-warmed plots of tallgrass prairie [33]. Above-ground biomass removal could significantly reduce C inputs from vegetation to soil and lead to significant N loss, resulting in substrate limitation to soil microorganisms [34], [35].

Understanding the fate of stored C and N and their potential for anthropogenic manipulation is critically important to evaluate the future state of the atmosphere or terrestrial ecosystems and manage the foreseen global change [36], [37]. However, the effects of management in relation to soil microorganisms on the redistribution and cycling of C and N within the plant-soil system were unclear. The objective of this study was to investigate the effects of compound fertilizer and clipping on C and N storage and distribution within a natural grassland ecosystem.

## Materials and Methods

### Site description and experimental design

The study was conducted in the National Dalaoling Forest Park near the dam of the Three Gorges Reservoir in China from July 2011 to September 2012. The experimental site located at approximately 110°56′E, 31°4′N and 1696 m asl. The climate in this region is of a northern subtropical type, with a warm, humid summer, and an obvious altitudinal change. Maximum, minimum, and mean annual temperature was 19.2°C in July, −2.7°C in January, and 8.5°C, respectively. The mean annual precipitation was 1446.8 mm. Although the majority of precipitation occurs in summer, there was still 179.6 mm in winter occurring as snow and sleet [38].

The experimental site had a vegetation coverage of more than 60%, composed of over 20 grass species, but was dominated by *Festuca arundinacea* Schreb (approximately 30% of total above-ground biomass), *Potentilla freyniana* (50% of total above-ground biomass) and *Lysimachia clethroides* Duby (10% of total above-ground biomass). In this humid ungrazed montane meadow, all grasses were shallow rooted in the depth of 0–20 cm, with the maximum density occurred in the 0–10 cm soil layer. The soil in the 0–20 cm depth zone had a pH of 5.8, 12.9 g organic matter per kg soil, 1.1 g total N per kg soil, and 0.4 g total P per kg soil.

The grassland was exposed to three treatments: (i) control; (ii) fertilization; and (iii) clipping. Five replicated plots were conducted for each of the three treatments and arranged in a randomized complete block design. Each plot was measured 10 m by 5 m and fenced on June 20, 2011 to prevent the rabbits or other animals from grazing. The grassland was untreated in the control. In the fertilization treatment, compound fertilizers (15-15-15, N-P_2_O_5_-K_2_O) were applied on July 15, 2011 and May 15, 2012 (600 kg per ha for each time). In the clipping treatment, the grassland vegetations were clipped to 3 to 5 cm with sickles on July 15, 2011 and May 15, 2012, respectively. The clippings were left *in situ*.

### Plant and soil sampling and analysis

Plant biomass was assessed five times from 2011 to 2012, in May (late spring 2012), July (middle summer 2011, 2012) and September (early autumn 2011, 2012). In each plot, five random 1 m×1 m quadrats were assigned. One quadrat was selected each time for plant sampling. Shoot including living and standing dead within the quadrat was collected. After the shoot was removed, the litter was picked up. Then, five soil sub-samples (7 cm in diam, and 0-5-10-20 cm depths) were collected from each quadrat using a soil auger and pooled together to be a composite sample. The roots (including roots and rhizomes) in the pooled soil cores were picked up and washed with deionized water three times to get rid of residual soil. Shoots, litters, and roots were killed at 105°C for 30 minutes and dried to constant weight at 80°C. The C and N concentrations of the plant samples of July 1, 2011 and July 18, 2012 were measured based on the methods described by Lu [39]. Each of the fresh composite soil samples of July 1, 2011 and July 18, 2012 was sieved (2 mm wire mesh) and divided into two sub-samples: one was kept in the refrigerator at 4°C until microbial analysis and the other was air-dried for the analysis of soil organic C and total N concentrations [39]. Plant and soil C, N storage was calculated as Post's and Tian's methods [40], [41].

### Soil microbial populations' analysis

Traditional culture techniques were used to determine the distribution of the main physiological groups [42]. Microbial populations of bacteria, fungi and actinomyces were determined by soil dilution plating on beef extract peptone medium, Martin's medium and Gause's No. 1 synthetic medium, respectively. All media were made up as the methods described by Dong et al. [43] and all microbes were cultivated in a 28°C incubator for 2, 3 and 7 d, respectively.

### BIOLOG analysis and calculation of microbial activity and diversity indices

The soil microbial activity and functional diversity was evaluated using BIOLOG ECO microplate (BIOLOG Inc., Hayward, CA, USA). Soil sample kept in the refrigerator (equal to 10.0 g dried soil) was suspended in 90 mL of sterile 0.85% NaCl solution, shaken at 220 rpm for 30 min and held for 5 min. Then the suspensions were diluted to a final dilution of 10^−3^ with sterile 0.85% NaCl solution. Each well of BIOLOG ECO plates was inoculated with 125 µL of the diluted soil extracts and incubated at 25°C. Optical density of the wells was read with BIOLOG Micro Station reader (MicroLog release 4.20) at 590 nm every 12 h for 7 days. The optical density readings were corrected for the water controls in subsequent analysis. Negative readings after the correcting were adjusted to zero. Soil microbial activity measured as AWCD was calculated by the method described by Garland and Mills [30]. The substrate richness, Shannon's diversity index, Shannon's evenness index, McIntosh's diversity index, and McIntosh's evenness index were calculated using the data at 72 h, since the highest rate of microbial growth was observed at this incubation time [44], [45]. Formulas used for the above indices calculations were described by Magurran [46] and Staddon et al. [47]. Principal component analysis (PCA) was performed on BIOLOG data divided by the AWCD [30].

### C and N sequestration rates

Changes of C (or N) sequestration were estimated by calculating the difference of C (or N) storage between July, 2012 and July, 2011. C and N sequestration rates (*CSR*, g C m^−2^ yr^−1^ and *NSR*, g N m^−2^ yr^−1^) were calculated using the following equations, respectively:
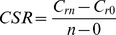
(1)

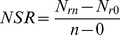
(2)where C_r0 and n_ is C storage (g C m^−2^) under the certain management (the fertilization, clipping or the control) during the first and second years in which C storage was measured, respectively; N_r0 and n_ is N storage (g N m^−2^) under the certain treatment (the fertilization, clipping or the control) during the first and second years in which N storage was measured, respectively; n is the number of years of duration of the experiment.

### Data analysis

Management effects and interactions between the variables were determined by the analysis of variance using SPSS 13.0 (SPSS, Inc.). Significantly different means were separated using Fisher's protected least significant difference (LSD) test (p<0.05). PCA analysis was performed using the Canoco 4.5 software package [48]. The data in July, 2011 were not shown since no obvious difference was found in effect of clipping and fertilization on soil microbial activity and diversity.

## Results

### Biomass

**Figure 1:**
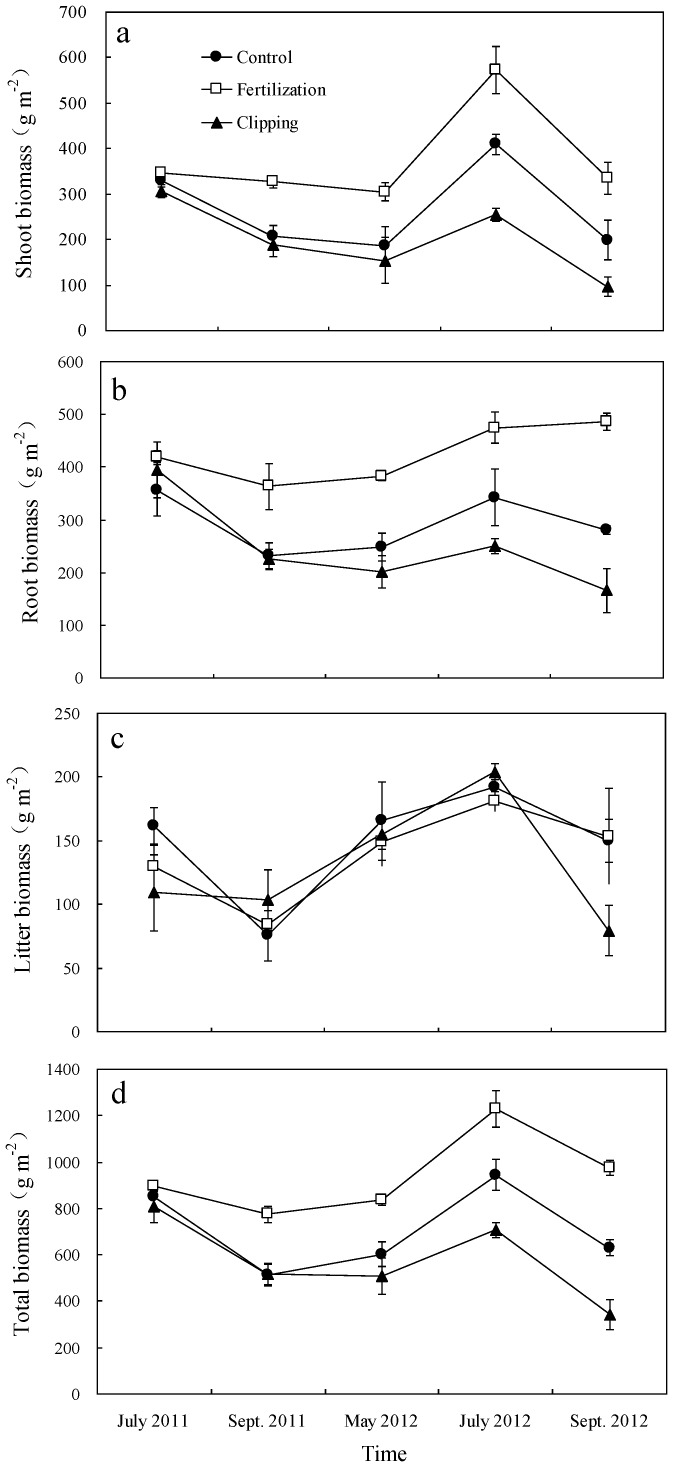
The shoot (a), root (b), litter (c) and total biomass (d) variation under the different treatments over time. Vertical bars represent standard error (SE). *n* = 5.

The shoot, root, litter and total biomass of the three treatments varied considerably through different growing season in 2011 and 2012 (Fig. 1). There was no significant difference in biomass among the treatments in July, 2011 (before the implementation of fertilization and clipping). As time went by, the fertilization treatment increased the shoot, root, and total biomass than the control. The clipping treatment, on the contrary, had the less shoot, root, litter and total biomass compared to the control. The total biomass was much higher in middle summer (July) relative to late spring (May) and early autumn (September) for each year. The total biomass in the fertilization treatment in July, 2012 was 30.1% and 73.4% greater than that in the control and clipping treatments, respectively.

### Plant and soil C, N storage and C: N ratios

Plant C storage ranged from 258.6 to 295.2 g C m^−2^, and soil C storage ranged from 2086.9 to 2752.5 g C m^−2^ for the three treatments as measured in July, 2011. Plant N storage ranged from 5.8 to 7.0 g N m^−2^, and soil N storage ranged from 199.8 to 223.0 g N m^−2^ for the three treatments as measured in July, 2011. No difference in C and N storage was found among the three treatments in July, 2011 (data were not presented).

**Figure 2:**
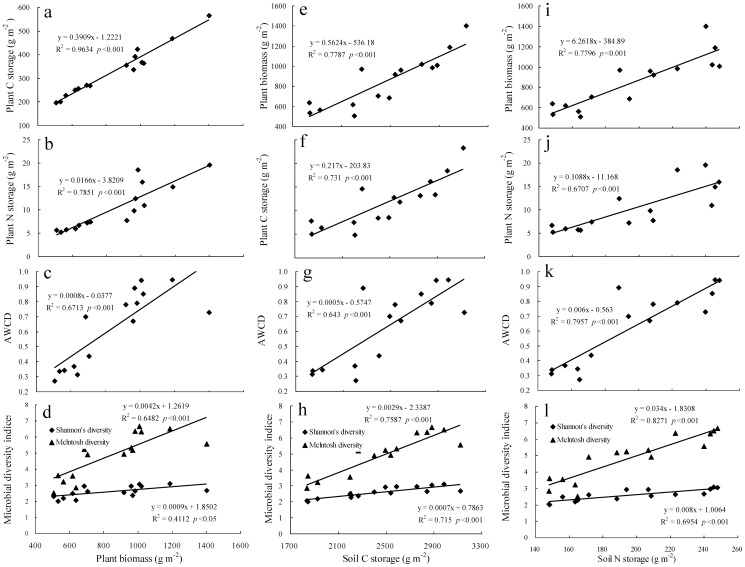
The relationships of plant biomass with plant C/N storage (a/b), AWCD (c) and soil microbial diversity indices (d), and the relationships of soil C/N storage with plant biomass (e/i), plant C/N storage (f/j), AWCD (g/k) and soil microbial diversity indices (h/l) across the three treatments in July, 2012.

The shoot and root C storage was the highest in the fertilization treatment, followed by the control and clipping in July, 2012 (Table 1). Shoot and root C storage in the fertilization treatment was 1.4 and 1.4 times more than that in the control, 2.2 and 1.9 times more than that in the clipping, respectively. Root C storage was similar between the control and clipping treatment. The shoot and root N storage of the fertilization treatment was 1.8 and 2.0 times more than the control, 3.0 and 2.8 times more than the clipping in July, 2012, respectively (Table 1). No difference in litter C and N storage was observed among the three treatments. The C: N ratios of all plant parts were decreased due to the fertilization treatment, but no difference was found between the clipping and the control (Table 1). Both plant C and N storage increased linearly as the plant biomass increased (both *p*<0.001; Fig. 2a, b).

**Table 1:** Carbon and nitrogen storage and C: N ratio among plant parts under different grassland treatments in July, 2012.

Treatments	Shoot	Root	Litter	Total
	––––––––C storage (g C m^−2^)––––––––			
Control	170.8±9.3b	139.3±21.8b	15.2±1.0a	325.2±24.1b
Fertilization	233.0±22.9a	195.8±19.3a	8.7±1.9a	437.5±37.0a
Clipping	108.0±7.1c	102.7±4.9b	16.0±2.6a	226.7±12.1c
	––––––––N storage (g N m^−2^) ––––––––			
Control	5.5±0.6b	2.8±0.5b	0.6±0.0a	8.9±1.0b
Fertilization	10.0±1.1a	5.6±0.9a	0.4±0.1a	16.0±1.5a
Clipping	3.3±0.1b	2.0±0.2b	0.5±0.1a	5.8±0.2b
	––––––––––C:N ratio––––––––––			
Control	32.1±2.2a	50.1±2.8a	26.7±1.5a	37.3±2.5a
Fertilization	23.6±1.9b	37.3±4.2b	20.3±1.8b	27.9±2.2b
Clipping	32.8±2.0a	51.5±2.0a	30.3±1.8a	38.8±1.1a

The data represent means ± SE (*n* = 5). Letters a, b, c in a column indicate statistical significance base on Fisher's protected LSD test (*p*<0.05) among the three different treatments.

Fertilized soil stored more C and N compared to the control and clipping treatments regardless soil depths in July, 2012 (Table 2). The soil had less C and N storage in the 0–5 and 10–20 cm zone in the clipping treatment. The fertilization increased soil C by 18.8% and soil N by 23.8%, respectively, when compared to the control. Whereas the clipping decreased soil C and N by 18.5% and 19.4% compared to the control, respectively. No significant difference in soil C: N ratio among the three treatments was found (Table 2). Both soil C and N storage increased linearly as the plant biomass increased (both *p*<0.001; Fig. 2e, i). Furthermore, the plant C storage increased linearly as the soil C storage increased (*p*<0.001; Fig. 2f), and the plant N storage increased linearly as the soil N storage increased (*p*<0.001; Fig. 2j).

**Table 2:** Carbon and nitrogen storage and C: N ratio of soils at the 0–5, 5–10, and 10–20 cm depths under different grassland treatments in July, 2012.

Treatments	0–5 cm	5–10 cm	10–20 cm	0–20 cm
	––––––––C storage (g C m^−2^) ––––––––			
Control	869.6±21.2b	573.1±26.7b	1014.1±47.5b	2456.9±56.5b
Fertilization	992.0±29.3a	732.9±39.1a	1193.8±29.5a	2918.8±62.5a
Clipping	690.3±29.1c	527.5±21.6b	784.4±52.4c	2002.2±82.7c
	––––––––N storage (g N m^−2^) ––––––––			
Control	64.8±5.3b	44.3±3.2b	84.2±2.0b	193.9±6.7b
Fertilization	79.6±2.5a	62.2±3.4a	98.3±1.5a	240.1±4.5a
Clipping	50.0±2.3c	40.6±2.1b	66.1±3.5c	156.2±3.6c
	––––––––––C: N ratio––––––––––			
Control	13.8±1.2a	13.1±0.9a	12.0±0.6a	12.7±0.4a
Fertilization	12.5±0.5a	11.9±0.9a	12.2±0.3a	12.2±0.3a
Clipping	13.9±0.9a	13.4±1.3a	11.9±0.4a	12.8±0.4a

The data represent means ± SE (*n* = 5). Letters a, b, c in a column indicate statistical significance base on Fisher's protected LSD test (*p*<0.05) among the three different treatments.

The high, medium and low values of total C and N storage (including plant and 0–20 cm soil zone) were observed in the fertilization, control and clipping grassland ecosystems, respectively (Tables 1, 2). The fertilization increased total C and N storage by 20.6% and 26.3%, respectively. The clipping reduced the total C and N storage by 19.9% and 20.1%, respectively. Furthermore, the plants had lower C and N storage and most C and N was stored in the soils. The 0–20 cm zone soils of the control, fertilization, and clipping treatments held 88%, 87% and 90% of the total C storage, and 96%, 94% and 96% of the total N storage, respectively.

### Soil microbial populations, activity and diversity

**Figure 3:**
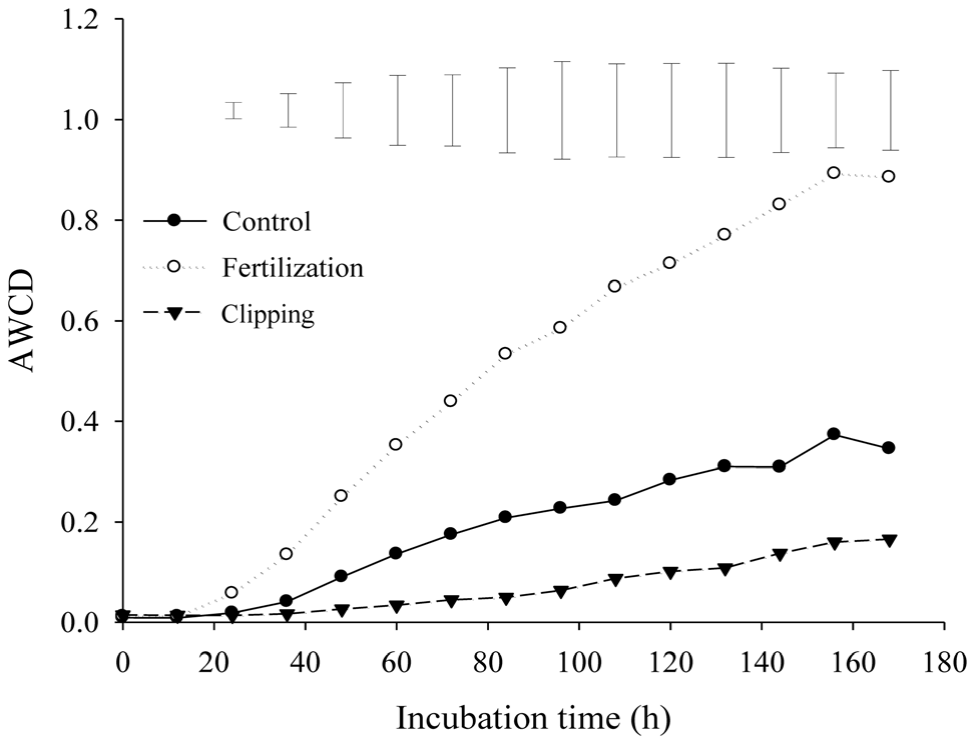
Change of average well color development (AWCD) of soil microbial community during the incubation time in July, 2012. Vertical bars represent Fisher's protected LSD (*p*<0.05).

**Figure 4:**
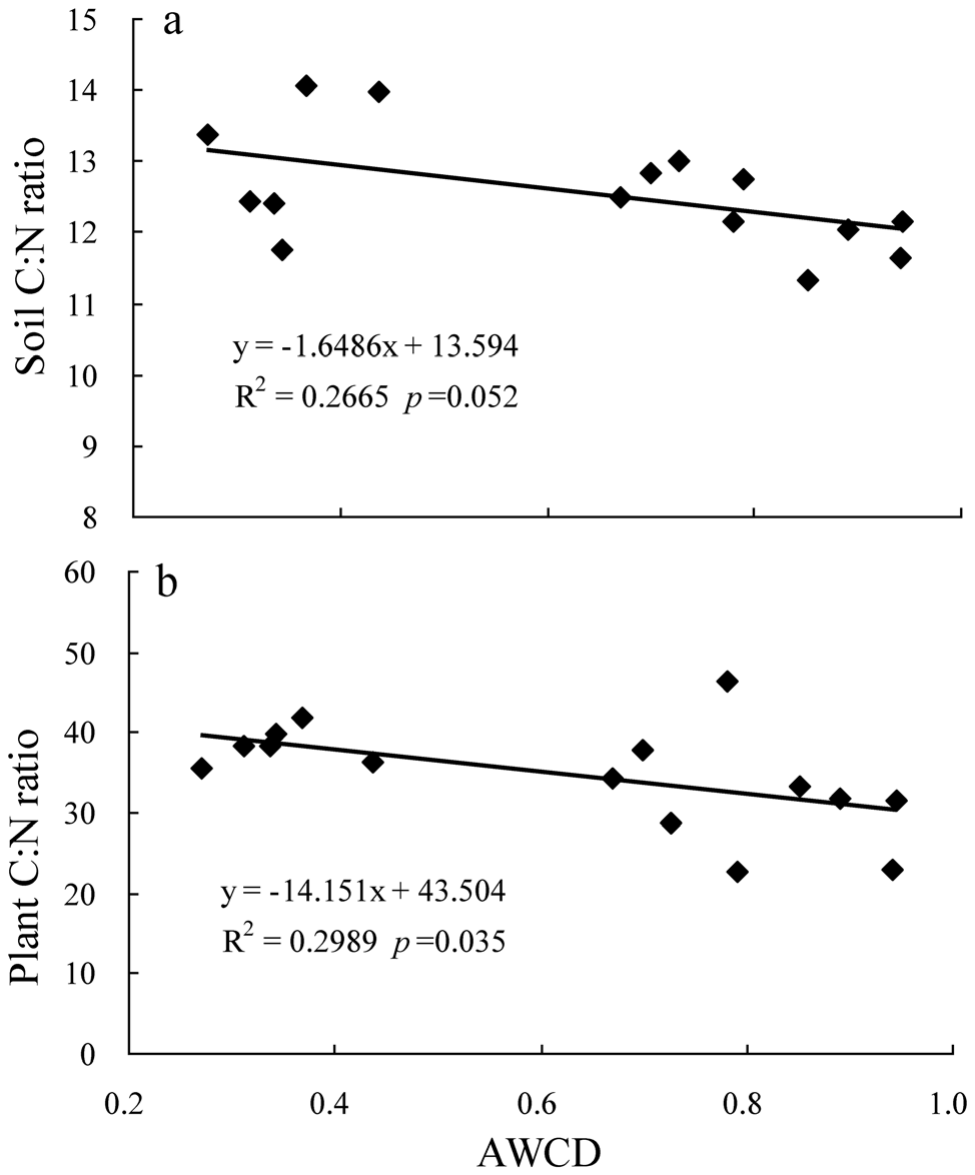
The relationships of AWCD of soil microbial community with soil C: N ratio (a) and plant C: N ratio (b) in July, 2012.

There was no difference in soil microbial number, activity and diversity among the three treatments in July, 2011 (data were not presented). On July 18, 2012, the number of bacteria, fungi and actinomyces in the fertilization treatment was 1.5, 1.3 and 1.6 times more than the control, and 2.3, 2.0 and 2.4 times more than the clipping treatment, respectively (Table 3). There was no difference in bacteria and actinomyces numbers between the control and the clipping. However, the clipping had fewer fungi than the control. The soil microbial activity (measured as AWCD) in July, 2012 was increased with incubation time for all the three treatments, and ranked in the order of fertilization > control > clipping (Fig. 3). The AWCD was positively associated with plant biomass, soil C and N storage (*p*<0.001, Fig. 2c, g, k). Both soil and plant C: N ratios decreased linearly with increasing AWCD (*p* = 0.052, *p* = 0.035 Fig. 4a, b). Soil microbial diversity, evenness indices and substrate richness in July, 2012 calculated from the BIOLOG data (72 h) were affected by the fertilization and clipping treatments (Table 4). The higher levels of substrate richness, Shannon's evenness index, and McIntosh diversity and evenness indices were detected in the fertilization treatment, but lower level in the clipping treatment, compared to that in the control. Both the Shannon's and McIntosh diversity indices were positively associated with plant biomass, soil C and N storage (*p*<0.001, Fig. 2d, h, l).

**Table 3:** The microbial community structure in grassland soils under different treatments in July, 2012. (CFU g^−1^ dry weight soil).

Treatments	bacteria number (×10^6^)	fungi number (×10^2^)	actinomyces number (×10^4^)
Control	2.8±0.1b	2.7±0.2b	1.2±0.1b
Fertilization	4.3±0.2a	3.6±0.1a	1.9±0.1a
Clipping	1.9±0.1b	1.8±0.1c	0.8±0.0b

The data represent means ± SE (*n* = 5). Letters a, b, c in a column indicate statistical significance base on Fisher's protected LSD test (*p*<0.05) among the three different treatments.

**Table 4:** Effects of fertilization and clipping on microbial functional diversity as evaluated by substrate richness (*S*), Shannon's diversity index (*H*′), Shannon's evenness index (*E (S)*), McIntosh diversity index (*U*) and McIntosh evenness index (*E (M)*) in July, 2012 (72 h).

Treatments	*S*	*H*′	*E (S)*	*U*	*E (M)*
Control	24.6±0.5b	2.7±0.1a	0.8±0.0b	5.1±0.1b	0.8±0.0b
Fertilization	27.0±0.6a	2.9±0.1a	0.9±0.0a	6.3±0.2a	0.9±0.0a
Clipping	21.8±0.4c	2.2±0.1b	0.7±0.0c	3.2±0.2c	0.5±0.0c

The data represent means ± SE (*n* = 5). Letters a, b, c in a column indicate statistical significance base on Fisher's protected LSD test (*p*<0.05) among the three different treatments.

**Figure 5:**
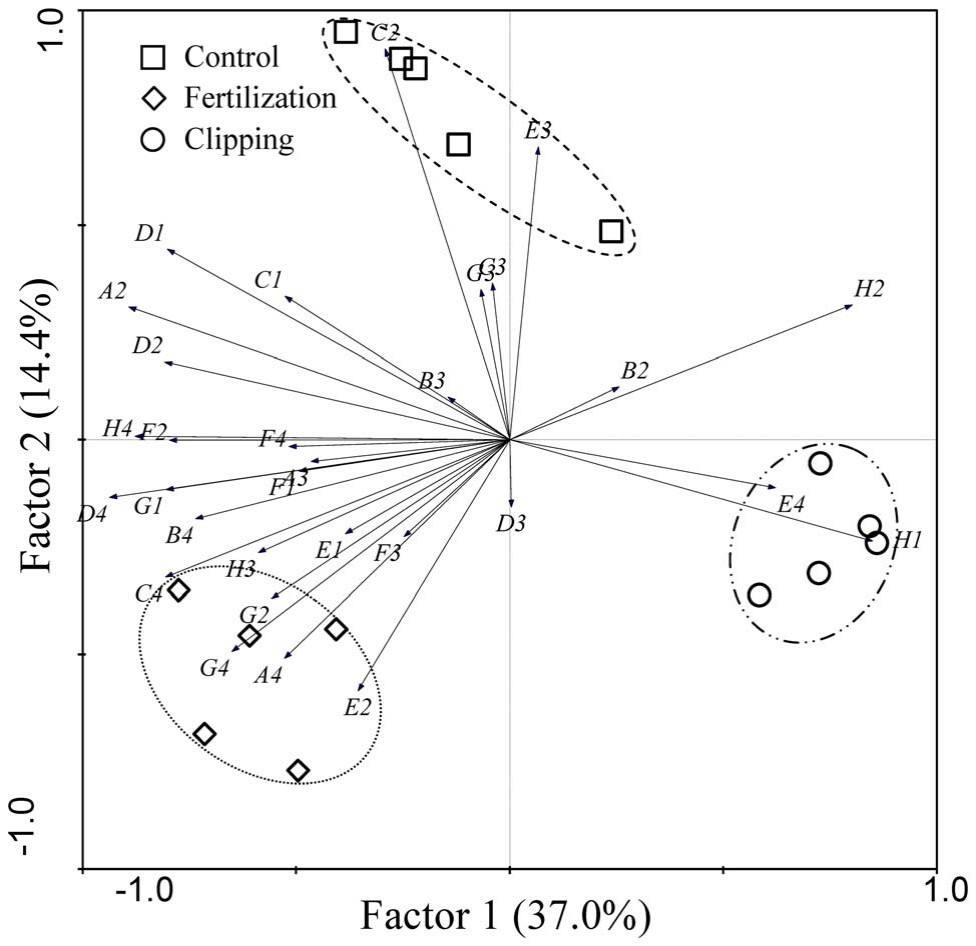
Principle components analysis of biological data in July, 2012 (72 h).

The PCA analysis indicated that the first two principal components accounted for 51.4% of the total variance (Fig. 5). The soil microbial community of the control, fertilization and the clipping treatments formed three distinct groups. The control was distinctly separated from the fertilization and clipping treatments by Factor 2. The fertilization and clipping treatments were distinctly separated by Factor 1. Furthermore, the factor loading plot also showed that the affinity of soil microbes for the substrates depended on the grassland treatments. The substrates including *L*-Arginine (A4), *L*-Phenylalanine (C4), N-Acetyl-D-Glucosamine (E2), Glucose-1-Phosphate (G2), Phenylethyl-amine (G4) and D-Malic Acid (H3) were favored by soil microbes of the fertilization treatment. The substrates including *L*-Threonine (E4), α-D-Lactose (H1), and D,L-α-Glycerol phosphate (H2) were favored by soil microbes of the clipping treatment, while the substrates including i-Erythritol (C2), 2-Hydroxy Benzoic Acid (C3), γ-Hydroxybutyric Acid (E3) and α-Ketobutyric Acid (G3) were favored by soil microbes of the control. Compared to the control, the fertilization treatment increased the utilization level of the substrates of amines (G4) and amino acids (A4 and C4), while the clipping treatment decreased the utilization level of the substrates of carboxylic acids.

### Plant and soil C, N sequestration rates

The plant, soil (0–20 cm) and total C, N sequestration rates during July, 2011 to July, 2012 were ranked in descending order of fertilization > control > clipping (Table 5). There was no statistical difference in the grassland soil (0–20 cm) and the total C and N sequestration rates among the three treatments. The plant C and N sequestration rates of the fertilization treatment were significantly higher than the clipping treatment (*p*<0.05).

**Table 5:** Carbon and nitrogen sequestration rates of plant and soil (0–20 cm) under different grassland treatments from July, 2011 to July, 2012.

Treatments	Plant	Soil (0–20 cm)	Total
	–––––C sequestration rate (g C m^−2^ yr^−1^) ––––––		
Control	66.6±33.6ab	9.0±328.3	75.5±229.3
Fertilization	142.3±43.3a	166.3±380.3	308.6±379.8
Clipping	−31.9±39.2b	−84.7±194.6	−116.6±510.3
	–––––N sequestration rate (g N m^−2^ yr^−1^) ––––––		
Control	2.9±1.0ab	−5.7±43.6	−2.9±43.8
Fertilization	9.0±2.1a	28.2±15.7	37.2±16.2
Clipping	−0.4±0.7b	−11.1±9.4	−11.5±10.0

The data represent means ± SE (*n* = 5). Letters a, b, c in a column indicate statistical significance base on Fisher's protected LSD test (*p*<0.05) among the three different treatments.

## Discussion

This investigation was conducted on the natural grassland with vegetation coverage of more than 60%, composed of over 20 species of grass. In grassland ecosystems, the immobilization of C and N in the soil is the basic solution for C and N sequestration. Schleinger [49] reported that the below-ground C pool generally had much slower turnover rate than above-ground C. The data collected on July 1, 2011 exhibited that soil C and N (i.e. 15 days prior to imposing experiment) was similar among the three treatments. However, soil C and N on July 18, 2012 (i.e. at the end of the two-year experiment) increased by 18.8% and 18.5%, respectively, in the fertilization relative to the control. Previous studies investigated the effect of fertilizer on the grassland C and N storage and indicated that the accumulation of soil C and N were attributed to the increase of plant biomass [50]–[52].

As the main source of soil organic matter, the increase of grass biomass (including the shoots, roots, senescent litter) may be the first step to enhance the C and N sequestration in the soil [10], [53]. The results of this study indicated that shoot and root biomass was greater in the fertilization treatment vs. the control, but less in the clipping treatment vs. the control. The increase in plant and soil C, N storage was significantly associated with a greater grass biomass (Fig. 2a, b, e, i). Data collected in July, 2012 indicated that the plant and soil C was significantly related to grass biomass (*r* ^2^ = 0.9634, 0.7787, both *p*<0.001). The correlation coefficient was 0.886 between plant N and biomass, and 0.883 between soil N and biomass (both *p*<0.001). Meanwhile, the plant C and N storage were positively associated with soil C and N storage (both *p*<0.001). Because the C and N stored in the plant was ultimately transferred into the soil in the forms of plant residues [54]. The beneficial effect of the fertilization on plant biomass could be contributed to the input nutrient [14], [55]. The compound fertilizer provided the essential elements of N, P, and K for plant growth, which improved the shoot, root, and total biomass. On the contrary, the clipping limited the plant growth by damaging photosynthesis organs, causing a slow-growing period and a decrease in grass biomass consequently [9]. In addition, the variation of grass biomass indicated that the biomass could be affected by seasonal variation, management types and management time. From the perspective of increasing biomass, spring fertilization could give better results compared with summer fertilization.

The nutrient for plant growth is mainly derived from decomposition of SOM and plant residues input to the soil [56]. Similar to previous studies [9], [57], we found that the plant of the fertilization treatment grew rapidly and sequestrated more C (carbohydrates) through photosynthesis and more N through passively and/or actively uptake, while the clipping treatment reduced the plant C and N uptake. Cheng et al [35] also found that the clipping decreased the plant N uptake in the tallgrass prairie. But Ruess et al [58] have reported that the clipping stimulated uptake rates of both ammonium (NH_4_^+^) and nitrate (NO_3_^−^), and ultimately accumulated more total plant N. This inconsistence was perhaps caused by the differences of soil nutrient conditions and plant species. The plant C: N ratio of the fertilization treatment was much lower than the control and the clipping treatments (Table 2). Chen [54] indicated that the SOM and plant residues with lower C: N ratios could be decomposed by microorganisms more easily due to improved soil microbial activity. We also found that the soil and plant C: N ratios decreased linearly with increasing AWCD (*p* = 0.035, *p* = 0.052). In addition, more humus was predicted for plant substrates with lower C: N ratios [59]. Thus the fertilization grassland could have a higher humification degree and sequestrated C and N in soil for a longer time than the control and the clipping treatments.

Soil microbial activity was involved in the mineralization of soil organic matter and plant residues [60], [61]. Previous studies [56], [62] indicated that the plant residues provided soil microorganisms with the major resource of nutrients and energy and controlled the soil microbial activity and composition. The results of this study exhibited that the fertilization and clipping treatments affected the grass biomass and ultimately changed the soil microbial activity, diversity and the C substrates utilized by soil microorganisms. The increase in soil microbial activity and diversity in the fertilization treatment was similar to Zhong and Cai's [29] and Marschner et al's study [14].

Our study suggested that the C and N cycles in the grassland ecosystem are determined not only by plant biomass, but also by soil microbial activity. The fertilization treatment, especially spring fertilization, improved the plant growth, increased the soil microbial activity, and ultimately increased the plant and soil C, N storage and sequestration rates. While the clipping treatment had the opposite effect and reduced the C, N storage and sequestration rates in grassland. Grassland management including fertilization was crucial to the grassland recovery from barren and overgrazing [63], [64]. Other studies [65], [66] indicated that the soil C may reach a new equilibrium in approximately decades after management changes, so continuous fertilization treatment was needed.

## Conclusions

The fertilization and the clipping treatments exhibited remarkable effect on the grass biomass, C and N storage, and soil microbial activity and diversity of grassland ecosystems in National Dalaoling Forest Park. After the two-year experiment, the compound fertilizer increased grass biomass, improved soil microbial activity and diversity and increased C and N sequestration in grassland ecosystem. The clipped plots had a lower level of C, N storage, which were mainly attributed to less grass biomass. The soil C, N storage was increased linearly with increasing grass biomass, plant C and N storage and AWCD. The principal component analysis indicated that the soil microbial communities of the control, fertilization and the clipping treatments formed three distinct groups, respectively. Previous and our results suggested that the fertilization might improve soil C and N slowly. So, to improve soil C, continuous fertilization management of grassland is needed. Long-term *in situ* studies to extrapolate the effect of the plant biomass on C and N dynamics combined to soil microbial activity and diversity and soil microclimate might contribute to a better understanding of C and N cycling and mitigation of global warming.
